# Semantic Clustering of Search Engine Results

**DOI:** 10.1155/2015/931258

**Published:** 2015-12-31

**Authors:** Sara Saad Soliman, Maged F. El-Sayed, Yasser F. Hassan

**Affiliations:** ^1^Department of Mathematics & Computer Science, Faculty of Science, Alexandria University, Alexandria 21511, Egypt; ^2^Department of Information Systems & Computers, Faculty of Commerce, Alexandria University, Alexandria 26516, Egypt

## Abstract

This paper presents a novel approach for search engine results clustering that relies on the semantics of the retrieved documents rather than the terms in those documents. The proposed approach takes into consideration both lexical and semantics similarities among documents and applies activation spreading technique in order to generate semantically meaningful clusters. This approach allows documents that are semantically similar to be clustered together rather than clustering documents based on similar terms. A prototype is implemented and several experiments are conducted to test the prospered solution. The result of the experiment confirmed that the proposed solution achieves remarkable results in terms of precision.

## 1. Introduction

Search engines are the main tool for searching and retrieving information from the Web. When the user query is submitted to traditional search engines, they return a list of results sorted in a way that depends on the search engine algorithm. However, while traditional search engines are useful for well-articulated search queries, they do not perform very well when it comes to ambiguous queries, which have more than one meaning. The result of an ambiguous query is typically large and diverse, making it hard for the typical user to analyze and comprehend. Comprehending such result might require the user to analyze a large result that normally contains irrelevant documents to reach for information of interest [1]. Such searches are known as “low precision searches” [2].

One way of helping users to find quickly what they are looking for is to group the search results by topics or categories. The process of grouping documents is called clustering, where grouping is applied to a set of documents so that documents belonging to the same cluster are similar and documents belonging to different clusters are dissimilar. Search results clustering can be defined as the process of automatically grouping results of the search into objective groups [2]. Systems that perform Web search results clustering, also known as* clustering engines*, have become popular in recent years [3]. Several commercial clustering engines have been launched recently; the most popular one among them is the Vivisimo engine [4]. Vivisimo won the “best meta-search engine award” assigned by Search Engine http://watch.com/ from 2001 to 2003 [3].

The main contribution of this work is introducing a new solution for clustering search engine result. Unlike most other search engine result clustering solutions, our solution does not just rely on the specific terms in the retrieved documents to compute similarities among documents and to perform clustering accordingly. Instead, proposed solution performs similarity comparisons and clustering based on the semantics of the retrieved documents. This is similar to what a human would do if asked to cluster a group of documents. This contributes largely to the quality of the resulting clusters as measured by the precision measure [5].

This paper is organized as follows.  Section 2 discusses related work.  Section 3 outlines the overall architecture of the proposed methodology.  Section 4 gives proposed experimental results. Finally, in Section 5, we conclude and describe our vision for the future work.

## 2. Related Work

Frequent Itemset Hierarchical Clustering (FIHC) [6] is a clustering technique of document which proposes the concept of the frequent item sets used in data mining. The idea of this technique is that documents which share a set of words that appear frequently are related, and this is used to cluster documents. This technique improves the scalability by reducing the dimensions by storing only the frequencies of the frequent articles which occur in a certain minimum fraction of the documents in vectors of document. TermRank [7] is a variation of the PageRank algorithm that counts term frequency not only by classic metrics of TF and TF × IDF but also by term-to-term associations. From each Web page the blocks in which the search keyword appears are retrieved. Suffix Tree Clustering (STC) [8] is a postretrieval document browsing technique (i.e., used in Grouper [9]). STC is an incremental and linear time clustering algorithm that is based on identifying the phrases that are common to groups of documents and building a suffix tree structure. Semantic, Hierarchical, Online Clustering (SHOC) [8] algorithm uses suffix arrays to extract frequent phrases and singular value decomposition (SVD) techniques to discover the cluster content. Lingo [10] combines common phrase discovery and latent semantic indexing techniques to group search results into meaningful groups. Lingo can create semantic descriptions by applying the cosine similarity equation and computing the similarity between frequent phrases and abstract concepts. The system presented in [11] consists of two separate phases. The first phase called “Indexing” builds an index to enable searching. The second phase called “Retrieval” allows users to submit queries and then uses the index to retrieve relevant documents. The result is clustered by using a Suffix Tree Clustering algorithm [8] and the user is presented with the clustering results.

Scatter/Gather [12] divides the data collection into a small number of clusters, the user selected clusters of interest, and the system reclustered the indicated subcollection of documents dynamically. Vivisimo [4, 13] is possibly the most popular commercial clustering search engine. Vivisimo calls search engines such as Yahoo and Google to extract relevant information (titles, URLs, and short descriptions) from the result retrieved. It groups documents in the retrieved result based on summarized information. The Vivisimo search clustering engine was sold to Yippy, Inc. in 2010. Grouper [9] uses* snippets* obtained by the search engines. It is an interface for the results of the Husky Search meta-search engine. Grouper uses the Suffix Tree Clustering (STC) algorithm to cluster together documents that have great common subphrases.* Carrot2* [14] is a clustering search engine solution that uses search results from various search engines including Yahoo, Google, and MSN. It uses five different clustering algorithms (*STC*,* FussyAnts, Lingo*,* HAOG-STC*, and* Rough k-means*) where Lingo Algorithm is the default clustering algorithm used. The output is a flat folder structure; overlapping folders are revealed when the user places the mouse over a document title. The system presented in [15] is a meta-search clustering engine, called the Search Clustering System (SCS), which organizes the results returned by conventional Web search engines into a cluster hierarchy. The hierarchy is produced by the Cluster Hierarchy Construction Algorithm (CHCA). Unlike most other clustering algorithms, CHCA operates on nominal data: its input is a set of binary vectors representing Web documents. Document representations are based either on snippets or on the full contents of the retrieved pages.

All of the above clustering engines except [15] use snippets that probably contain terms that are part of the query keywords. Snippets are not necessarily good representative of the whole document contents, which affects the quality of the clusters. Proposed solution uses whole documents rather than titles and short snippets, to ensure proper extraction of the semantics of the retrieved documents. While all of the above clustering engines have been mostly performed without explicit use of lexical semantics, proposed work takes into consideration both lexical and semantics similarities. This enables proposed system to provide better clustering quality.

## 3. Overall Architecture of the Proposed Methodology

**Figure 1:**
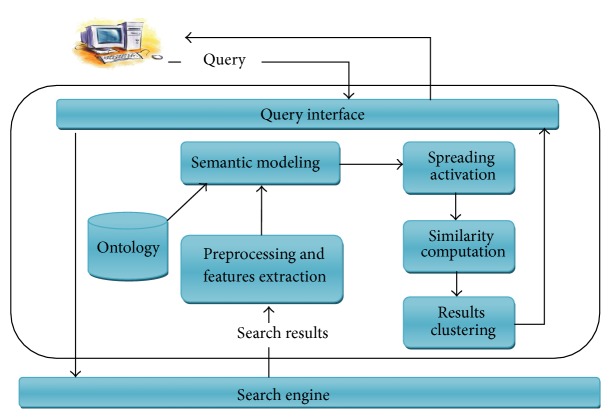
System overview.

The framework of the proposed solution is shown in Figure 1. The system receives the user's query (*q*) which is expressed in terms of keywords. The system performs the following steps:

- Submitting the query to a search engine and receiving the result.
- Preprocessing documents from the results and extracting features from each document.
- Enriching document features using ontology and constructing semantic network to model the document.
- Applying spreading activation algorithm on the constructed semantic network.
- Computing the dissimilarity matrix among documents using the most significant features representing the retrieved documents as highlighted by spreading activation.
- Applying clustering algorithm on the similarity matrix to obtain the clusters.

Now, we will describe in detail each of the steps of proposed solution using a simple example to elaborate the semantic clustering ability of our proposed solution which sets it apart from term-based clustering solutions.

Four simple documents are used: *D*1, *D*2, *D*3, and *D*4. Each of these four documents has only two terms (with frequency one for each of them). After preprocessing the documents and extracting the features, we got the following:

(1) D1=Apple: 1,Headphone: 1,D2=Apple: 1,diet: 1,D3=Orange: 1,diet: 1,D4=Samsung  galaxy: 1,Bluetooth: 1.

The similarity matrix between the four documents is formed using extracted terms in the same way as in the traditional clustering approaches as shown in ∗.

*Similarity Matrix among the Four Documents*. Consider
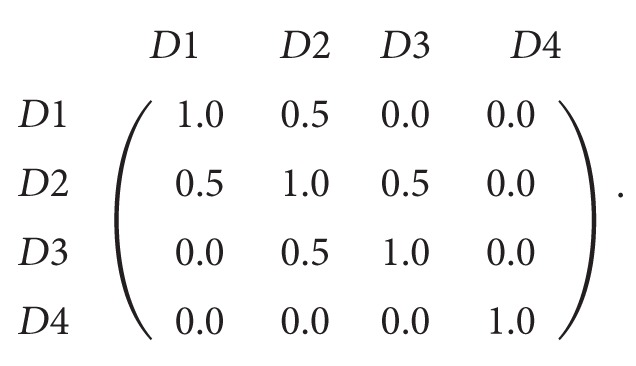
(∗)If we cluster the four documents using the computed similarly values in ∗ we get the following clusters:

(2) C1=D1,D2,D3,C2=D4.

When we examine the term-based similarity values as shown in ∗, we found that it is not acceptable from the perspective of the human being because of the following reasons:

(i) While *D*1 and *D*2 are two documents that are reflecting two totally different domains the computed similarity value is 50%.
(ii) The similarity value between *D*1 and *D*4 is 0% while both documents are related to mobile phones.
(iii) The similarity value between *D*2 and *D*3 is only 50% while both documents are related to health and diet.

Then the resulting clusters *C*1 and *C*2 have very low precision, 0% for this simple example.

Now we will discuss how proposed solution computes the similarity and clusters for the same four documents.

### 3.1. Preprocessing and Feature Extraction

**Figure 2:**
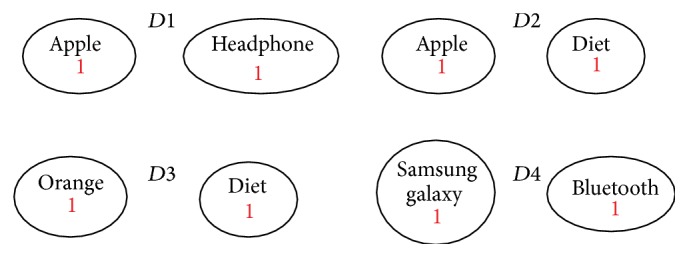
Initializing the graphs for the four documents.

The proposed solution first extracts terms from each retrieved document through tokenization and then removes stop words (e.g., a, on, be, as…) from the token set. Multiword phrases were taken into consideration. Next, lemmatized terms are used based on the WordNet [16]. Finally, the proposed solution initializes graph *V* using the extracted features. Each feature becomes a node in *V* and is annotated with the frequency of the term in *d*_*i*_.  Figure 2 shows the initialization of the four graph nodes representing the four documents in our running example.

### 3.2. Feature Enrichment

**Figure 3:**
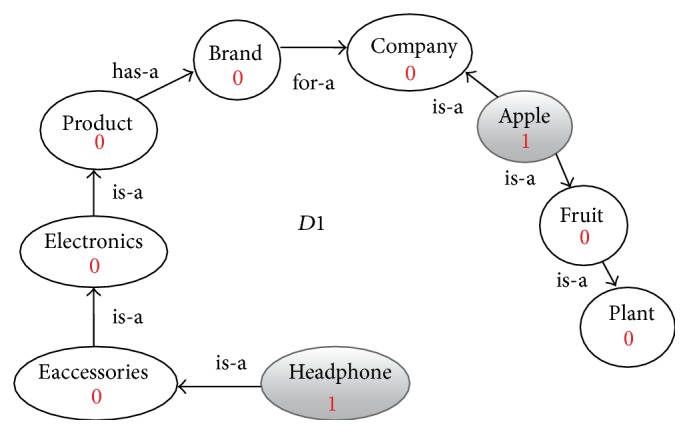
Enriched graph for *D*1.

**Figure 4:**
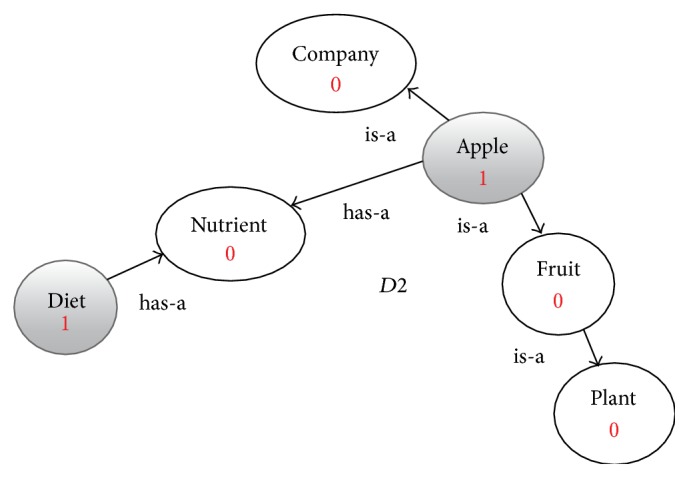
Enriched graph for *D*2.

**Figure 5:**
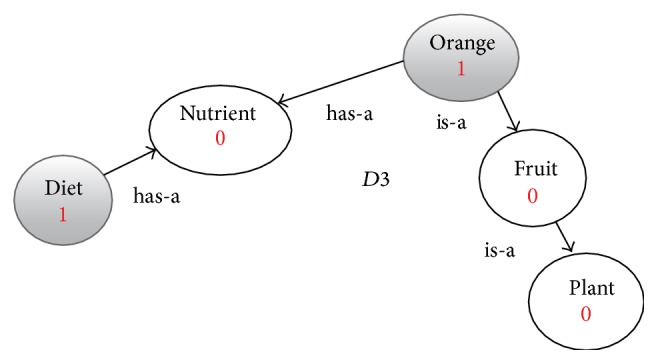
Enriched graph for *D*3.

**Figure 6:**
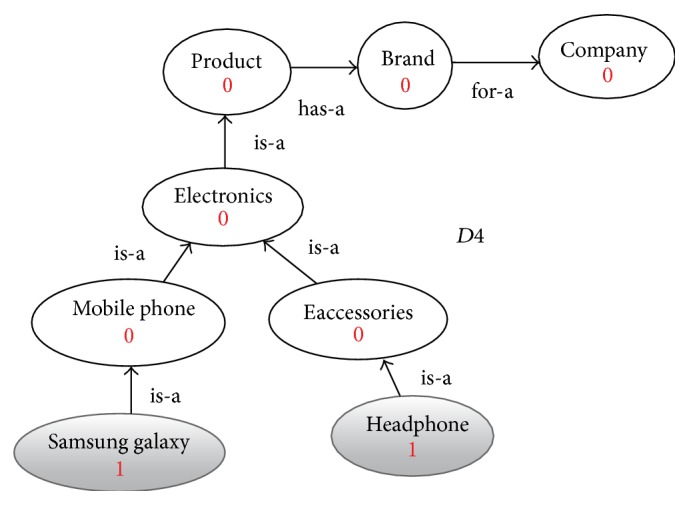
Enriched graph for *D*4.

Using the ontology, proposed solution augments *V* with concepts and relationships from ontology, which are related to terms in *V*. This process enriches *V* both lexically and semantically. The concepts that are added to *V* are assigned frequency of zero. Unlike many other semantic systems that rely mainly on WordNet, our system uses ontology that contains not only lexical terms and relationships but also other semantic terms and relationships. Our ontology can be depicted in the form of an enriched graph that could be considered as a semantic representation of the retrieved document. In this graph, terms with similar meaning represent a concept. Figures 3, 4, 5, and 6 show the enriched graphs.

### 3.3. Spreading Activation

The graph that we have constructed in the last steps not only models the retrieved document but also contains additional lexically and semantically relevant concepts and relationships. These additional concepts and relationships help in linking features of the retrieved document. The enriched graph does not yet contain enough semantics to perform high quality clustering because of two reasons:

- Concepts and relationships that are added to the graph are mainly related to the terms in the original document and not filtered to the specific semantics of the document. Hence, they could be contained in a graph representation of another document that has similar terms even if this document has different semantics.
- The weight of the added concepts is initially set to zero, which does not reflect their relative importance of those concepts to the semantics of the document.

Proposed solution resolves the two issues above through two steps:

(a) Selecting of concepts and relationships in *V* that is semantically relevant to the context of the document. This is done through applying a shortest path algorithm [17], shown in Algorithm 1.
(b) Adjusting the weights of concepts in *V* to better integrate new information that is added to *V*. This will affect the similarity computation in later step. We perform that through a spreading activation process [18].

These two steps allow proposed solution to perform semantic clustering rather than term-based clustering. This leads to better clustering result as will be shown in the experiments section.

**Figure 7:**
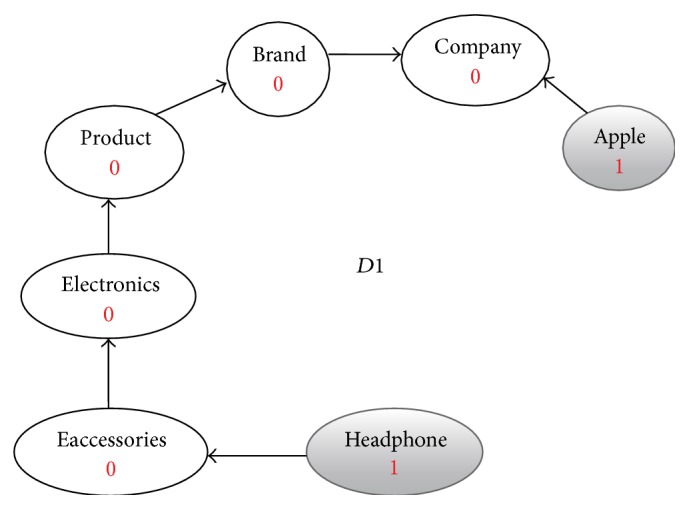
Shortest path for *D*1.

**Figure 8:**
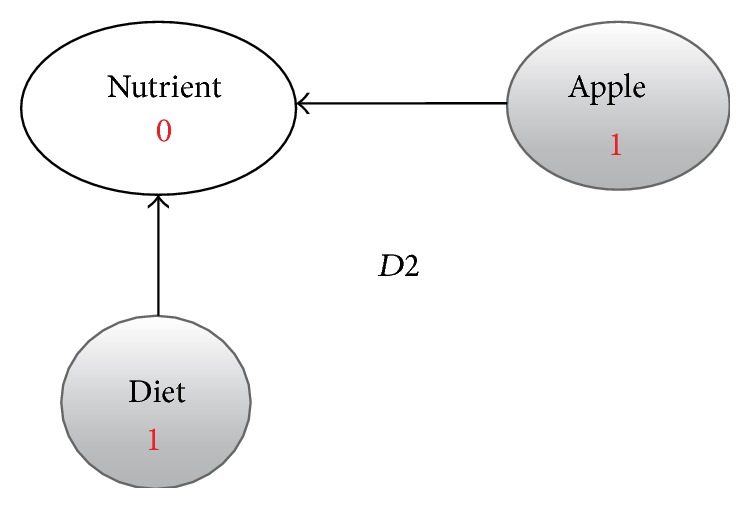
Shortest path for *D*2.

**Figure 9:**
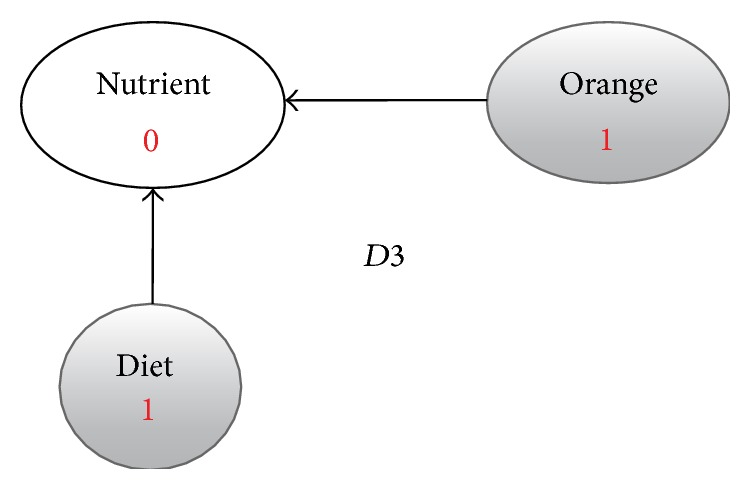
Shortest path for *D*3.

**Figure 10:**
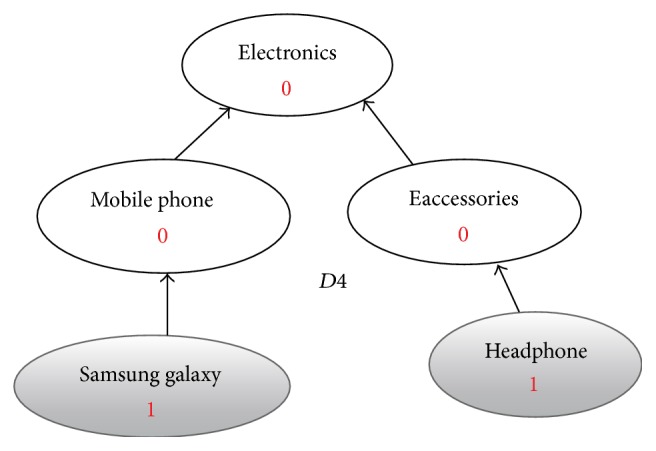
Shortest path for *D*4.

Proposed system computes the shortest path using the Floyd-Warshall algorithm [17]. The pseudocode of shortest path procedure is shown in Algorithm 1. Figures 7, 8, 9, and 10 show the nodes and relationships in shortest path for every graph.

After determining nodes and relationships in the shortest path, our system applies a spreading activation algorithm [18] that operates on nodes in the shortest path. We consider the frequencies on the graph nodes as initial activation values for the spreading activation process.

The main idea of spreading activation is to activate nodes and to propagate this activating from one node to other nodes while incrementing frequencies. The pseudocode of the spreading activation procedure that proposed solution uses is shown in Algorithm 2. The algorithm consists of the following steps:

(i) Initial nodes to be activated are placed in a priority queue.
(ii) The current node spreads its activation value to its neighbors. Considering the source node as *i* and the target node as *j*, spreading to the neighbors occurs according to (3), where *I* denotes input and *O* denotes output:
  (3) $$\begin{array}{c} I_{j}\left(\;t+1\;\right)=I_{j}\left(\;t+1\;\right)+O_{i}\left(\;t\;\right)*w_{ij}. \end{array}$$
(iii) The contribution of *i* is added to the current input value of node *j*. Thus, the algorithm rewards those nodes which are reached through different paths, by adding the contributions of all its neighbors. This contribution is obtained by multiplying the output value of node *i* (*O*_*i*_(*t*)) by the weight of the edge *w*_*ij*_.
(iv) The output of a node is given by the function *O*_*i*_(*t*).

The value *w*_*ij*_ corresponds to the numerical weight of the relationship obtained from the ontology proposed solution uses; in running example the weight is equal to 1. At the end, the result list contains the nodes which represent the result of the spreading activation process.

**Figure 11:**
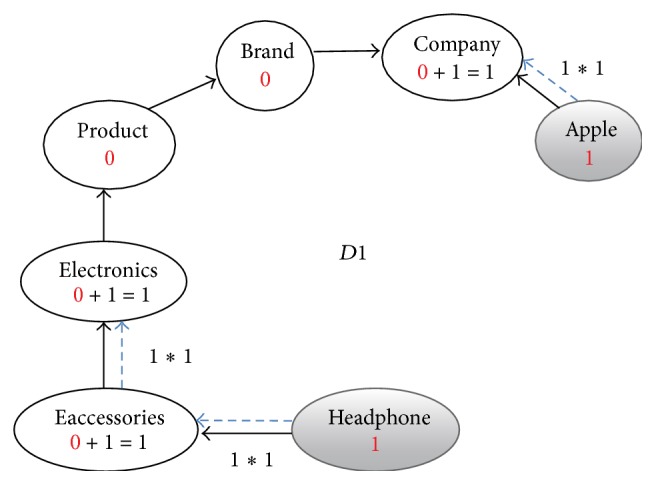
Spreading activation for *D*1.

**Figure 12:**
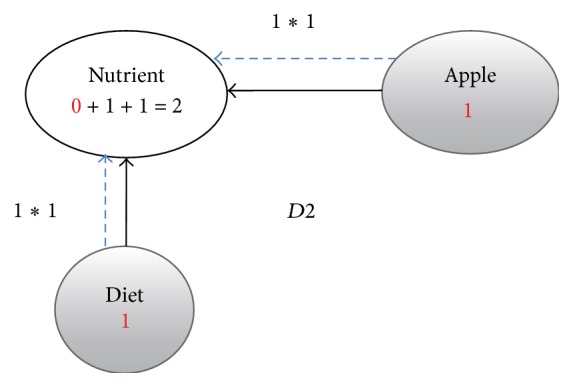
Spreading activation for *D*2.

**Figure 13:**
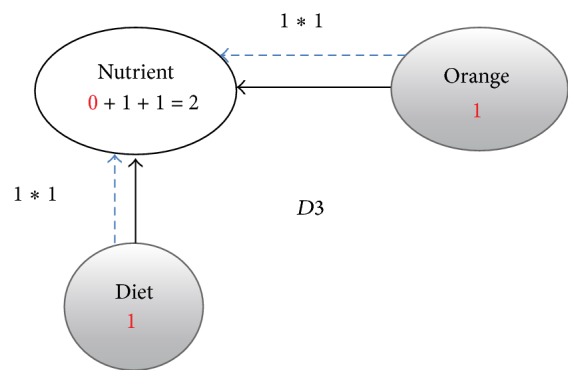
Spreading activation for *D*3.

**Figure 14:**
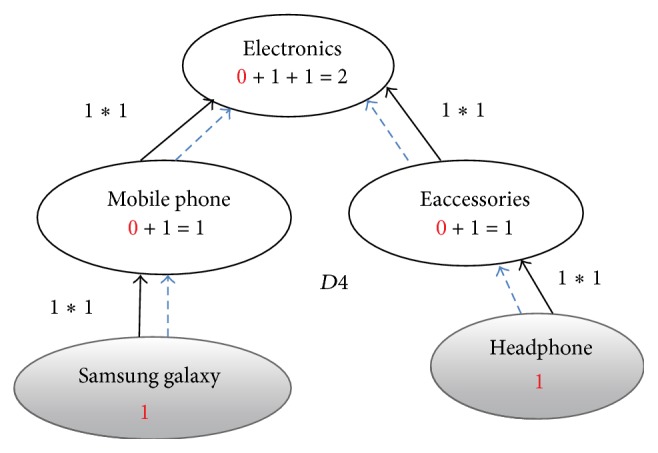
Spreading activation for *D*4.

Figures 11, 12, 13, and 14 show the execution of the spreading activation algorithm for the four examples.

**Figure 15:**
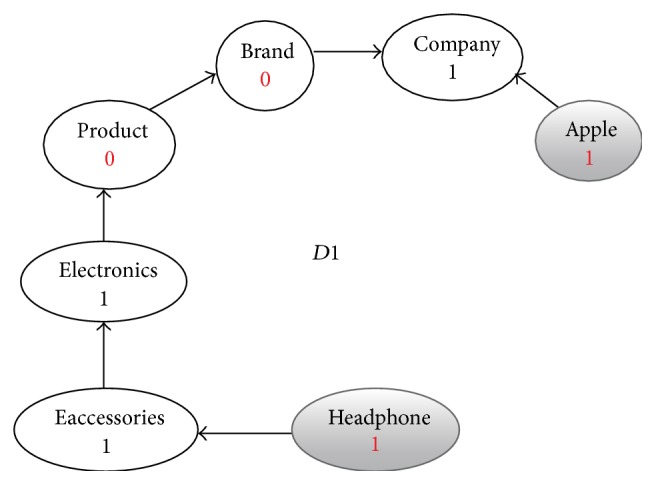
Frequencies after spreading activation for *D*1.

**Figure 16:**
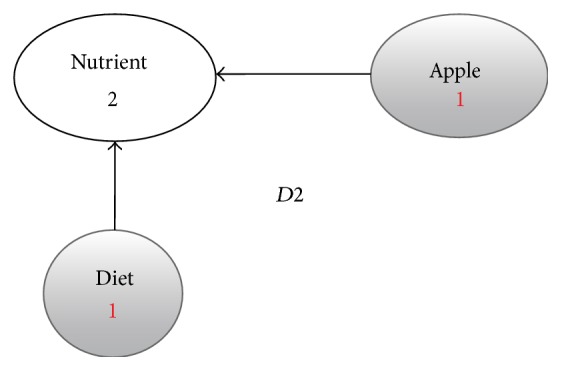
Frequencies after spreading activation for *D*2.

**Figure 17:**
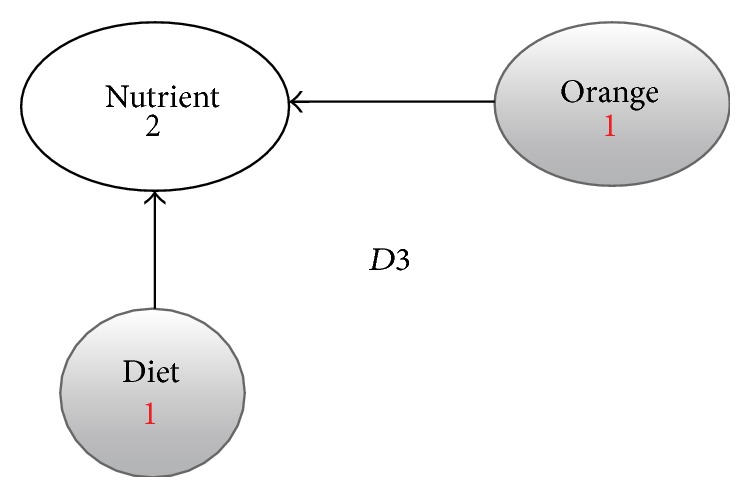
Frequencies after spreading activation for *D*3.

**Figure 18:**
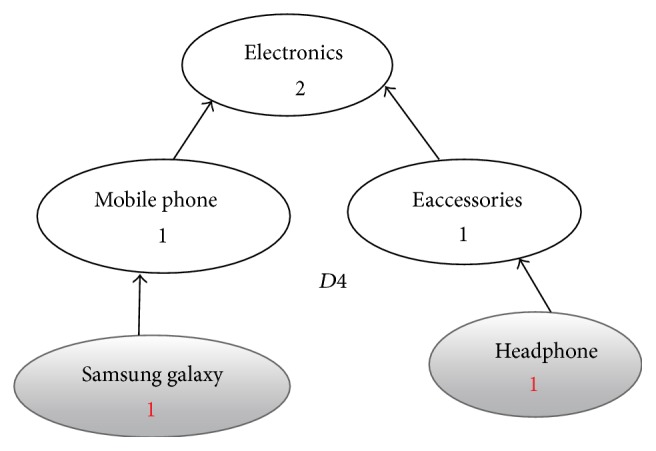
Frequencies after spreading activation for *D*4.

The final frequency values of nodes in the four graphs after spreading activation are shown in Figures 15, 16, 17, and 18.

### 3.4. Similarity Computation

After applying the shortest path algorithm and the activation spreading algorithm, the concepts with their frequencies in each semantic network graph are extracted to be used in the similarity comparison between every two documents.

Proposed solution uses the cosine similarity function [19] to check the similarity between the extracted concepts representing two documents. The cosine similarity function is shown in

(4) $$\begin{array}{c} sim\left(\;s,d\;\right)=\frac{\sum_{t\in \left(s,d\right)}w_{s}\left(t\right)\cdot w_{d}\left(t\right)}{\sqrt{\sum_{t\in s}w_{s}{\left(t\right)}^{2}}\cdot \sqrt{\sum_{t\in s}w_{d}{\left(t\right)}^{2}}}, \end{array}$$

where *s*, *d* are the two documents, *w*_*s*_(*t*) is the weight of term *t* in the *s* document, and *w*_*d*_(*t*) is the weight of term *t* in the *d* document.

Equation ∗∗ shows the calculated similarity between every two documents based on the features and frequencies obtained from our solution.

*Similarity Matrix as Computed by Proposed Solution*. Consider
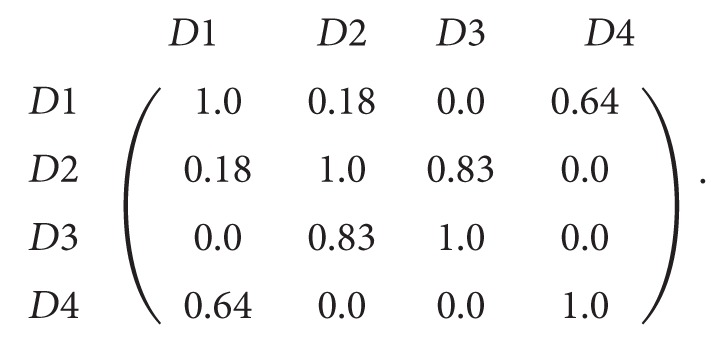
(∗∗)


The similarity values computed by proposed solution, shown in ∗∗, reflect better results according to the semantics of the documents. In particular, consider the following:

(i) *D*1 and *D*2 similarity is 18% in our solution instead of 50% in term-based solutions.
(ii) *D*1 and *D*4 similarity is 64% in our solution instead of 0% in term-based solutions.
(iii) *D*2 and *D*3 similarity is 83% in our solution instead of 50% in term-based solutions.

### 3.5. Results Clustering

Proposed solution uses an agglomerative hierarchical clustering [20], which is a bottom-up clustering method. The Euclidean distance that was used is the similarity measure. In initialization, each document is considered as cluster. Similar documents are merged into cluster until a termination condition is satisfied. This condition could be reaching a certain number of clusters (*k*). The agglomerative hierarchical clustering algorithm is shown in Algorithm 3.

If proposed solution in this case clusters the four documents using the computed similarly values in ∗∗, the solution gets the following clusters:

(5) C1=d1,d4,C2=d2,d3.

This clustering result has very high precision, 100% for this simple example, and has major improvement over the result as shown above when traditional clustering has been performed.

## 4. Experimental Results

A prototype was built for testing proposed solution using Java programming language. The prototype performs search engine result preprocessing, feature extraction and modeling, ontology enrichment, spreading activation, and similarity computation. Protégé [21] is used for building the ontology which has been used in the experiments. We use Jena [22] as an API programmatic environment for querying RDF and OWL based data models that uses the SPARQL query language [23, 24]. The agglomerative clustering algorithm was implemented using R software [25].

To compute the quality of the result we use the precision measure which represents the percentage of positive predictions by the system that is correct as shown in (6). We use human clustering as the reference for the correctness of the result clustering, where three different people conducted the manual clustering and the results obtained from them were validated:

(6) PCj=CjtCj,P=∑Cj∈CPCjCj∑CjCj.

We have used ten different queries for testing, namely, “Apple,” “Paris,” “Jaguar,” “Hollywood,” “Red Hot Chili Peppers,” “Mac,” “Snow Leopard,” “Lion,” “Tiger,” and “Mouse.” The first 5 queries were also used for testing in [11]; thus we use them for comparison purposes.

We ran two experiments; the first one measures the precision of the clustering resulting from our solution when applying all phases of the solution. The second experiment tests the system without applying the spreading activation step to determine the significance of spreading activation. In our experiments we limit documents to be clustered from the result to 20 documents for each query and we set numbers of clusters to 5. We use https://www.google.com/ as the search engine of choice for retrieving results related to our experiments.

Table 1 shows precision values for the resulting clusters in first experiment.

The results reported in [11] for the first 5 queries are 57.5% for the query “Apple,” 85% for the query “Paris,” 76% for the query “Jaguar,” 86% for the query “Red Hot Chili Peppers,” and 86.5% for the query “Hollywood.”

In the second experiment (running the system without spreading activation), the resulting precision values are shown in Table 2.

Comparing these values to the values obtained in experiment 1, we conclude that activation spreading has contributed to large extent to the high precision results of our solution.

As explained earlier, the spreading activation algorithm step gives the proposed solution the ability to perform similarity comparison and to cluster the document on the semantic level rather than on the syntax level, which sets the proposed solution apart from most other solutions. This allows the proposed solution to function in a way that is similar to a large extent to what a human will do if asked to cluster the documents.

## 5. Conclusion

Searching the Web is a task that consumes too much time and effort especially for ambiguity queries which have many meanings. WebPages clustering could help in reaching the required documents that the user is searching for. In this paper a novel approach has been introduced for search results clustering that are based on the semantics of the retrieved documents rather than the syntax of the terms in those documents. This means that documents that are semantically similar are clustered together rather than clustering together documents that just contain similar terms. The proposed solution has been implemented and tested. Our experiments show remarkable accuracy level for our solution. Our future work is to examine the effect of using more constraints in the spreading activation step, scaling the solution to support large number of retrieved search engine results and improving the ontology used to support more queries and domains.

## Figures and Tables

**Algorithm 1:**
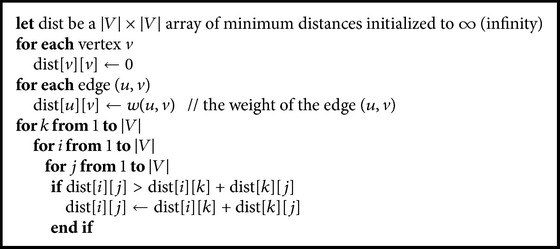
Shortest path.

**Algorithm 2:**
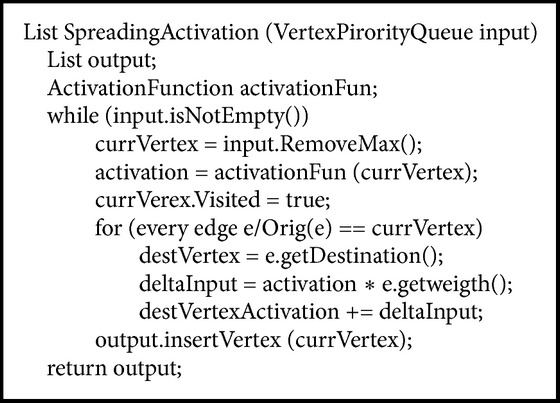
Spreading activation.

**Algorithm 3:**
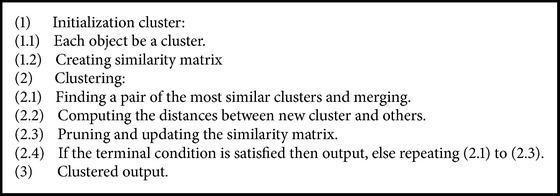
Clustering algorithm.

**Table 1:** The first experiment values.

Query	Precision
Apple	95%
Paris	90%
Jaguar	90%
Hollywood	95%
Red Hot Chili Peppers	95%
Mac	85%
Snow Leopard	90%
Lion	80%
Tiger	85%
Mouse	95%

**Table 2:** The second experiment values.

Query	Precision
Apple	75%
Paris	80%
Jaguar	60%
Hollywood	40%
Red Hot Chili Peppers	75%
Mac	75%
Snow Leopard	80%
Lion	70%
Tiger	60%
Mouse	85%
